# Co-developing and evaluating the acceptability of a Dyadic mind-body intervention for Relieving Enduring sleep disturbances Among older individuals with deMentia and their caregivers (DREAM) across White and ethnic minority communities in the UK

**DOI:** 10.1192/j.eurpsy.2026.11995

**Published:** 2026-07-31

**Authors:** S. Chan, J. Selwood, S. Popel, R. Cheston

**Affiliations:** 1 Centre for Health and Clinical Research, University of the West of England; 2 Bristol Dementia Wellbeing Service; 3 School of Social Sciences, University of the West of England, Bristol, United Kingdom

## Abstract

**Introduction:**

Sleep disturbance is common in dementia and contributes to the pathogenesis and progression of Alzheimer’s disease, while also increasing caregiver burden. Mind–body approaches look promising, but acceptability in culturally diverse, dyadic formats is under-examined. Co-development with communities may enhance cultural relevance and uptake.

**Objectives:**

To co-develop and culturally adapt the DREAM programme with White British, Caribbean, Chinese and South Asian stakeholders; to evaluate acceptability among people with mild dementia and caregivers; and to generate behavioural insights for implementation.

**Methods:**

A mixed-methods co-design employed Intervention Mapping (IM; Stages 1–4) alongside the COM-B model (Capability, Opportunity, Motivation → Behaviour). Stakeholders included 12 dementia–caregiver dyads, PPI representatives, mind-body practitioners, clinicians, and community leaders. Stage 1 synthesised focus groups/interviews and a scoping review, coded with COM-B. Stages 2–3 produced change-objective matrices and mapped strategies to education, training, and modelling, specifying techniques such as action planning and habit formation. Stage 4 defined an eight-week dyadic programme—gentle Tai Chi, brief mindfulness, and home practice—culturally tailored. Acceptability was assessed via session ratings, engagement (attendance, home practice), and semi-structured interviews. Data were integrated using a convergent design.

**Results:**

Co-design produced a logic model, change matrices, a facilitator manual and culturally adapted materials. COM-B analysis highlighted capability needs (simplified movements, shorter practices, step-by-step guidance), opportunity enablers (trusted community venues, flexible scheduling, caregiver-supported home practice) and motivation drivers (perceived dyadic benefit, sleep goals, cultural resonance). Session ratings and feedback indicated good acceptability; many participants adopted brief bedtime routines. Engagement indicators were favourable (regular attendance and frequent home practice), with no safety concerns. Interviews underscored coherence, adaptability and relative advantage over usual advice, while noting barriers (competing caregiving demands, transport, language preferences) and supports (translated handouts, facilitator diversity, train-the-trainer pathway).

**Conclusions:**

IM combined with COM-B enabled systematic, culturally responsive co-development of DREAM. Early evidence suggests good acceptability across White and ethnic minority communities and identifies behavioural targets to optimise engagement and delivery. Findings support progression to feasibility testing (IM Stages 5–6) to assess recruitment, fidelity and preliminary sleep outcomes, alongside implementation planning for scale-up.

**Disclosure of Interest:**

None Declared

